# Clinicopathological characteristics and survival outcomes in pleomorphic lobular breast carcinoma of the breast: a SEER population‐based study

**DOI:** 10.1002/cam4.1244

**Published:** 2017-11-13

**Authors:** Li‐Peng Yang, He‐Fen Sun, Yang Zhao, Meng‐Ting Chen, Nong Zhang, Wei Jin

**Affiliations:** ^1^ Department of pathology School of Basic Medical Sciences Fudan University Shanghai 200032 China; ^2^ Department of Breast Surgery Key Laboratory of Breast Cancer in Shanghai Collaborative Innovation Center of Cancer Medicine Fudan University Shanghai Cancer Center Shanghai 200032 China; ^3^ Department of Oncology Shanghai Medical College Fudan University Shanghai 200032 China

**Keywords:** Invasive ductal carcinoma, pleomorphic lobular cancer, SEER, survival outcomes

## Abstract

The purpose of this study was to explore the clinicopathological features and survival outcome of pleomorphic lobular carcinoma (PLC) of breast, we identified 131 PLC patients and 460,109 invasive ductal carcinoma (IDC) patients in the Surveillance, Epidemiology, and End Result (SEER) database. PLCs presented with increased lymph node involvement, older age, higher AJCC stage and grade, and lower median survival months (PLC 84 ± 51.03 vs. IDC 105.2 ± 64.39 *P* < 0.01). Compared to IDC patients, PLC patients were more inclined to be treated with mastectomy. In univariate analysis, PLC patients showed a worse disease‐specific survival (DSS) than that of IDC patients (hazard ratio = 0.691, 95% confidence interval 0.534–0.893, *P* < 0.01). In multivariate analysis, we took into account other prognostic factors and found that the histology types were no longer an independent prognostic factor (*P *= 0.120). DSS have no difference between matched IDC and PLC groups (*P* = 0.615). This result may be due to PLCs presenting higher tumor stage, higher tumor grade, and higher rate of LN metastasis than IDCs. Our conclusion is that PLC and IDC have many different characteristics, but there is not enough difference on the DSS.

## Introduction

Page and Anderson first described PLC in 1987 1, Eusebi et al. 2 and Weidener 3 consolidated the histological features. PLC is a rare form of invasive lobular carcinoma (ILC) with important clinical values 4. PLC has the specific loosely cohesive growth pattern of ILC and shares molecular alterations with ILC, such as the alterations of gene CDH1 5, however, compared with ILC, PLC has its own unique characteristics such as more frequent mitotic figures, increased hyperchromatic, and a single prominent nucleolus etc. 5. PLC has been shown to be more commonly represented in BRCA2 carriers 6.

IDC is a group of malignant epithelial tumors that tend to invade adjacent tissues and metastasize to distant sites 7. Nuclear atypia and pleomorphism of IDC are consistent with PLC 8. The biological aggression of PLC is related to the genetic alterations of high‐grade ductal carcinoma, such as overexpression of c‐myc and HER2/neu5. Monhollen and Middleton 9, 10 suggested that PLC carried a higher risk of metastasis and recurrence than IDC. They also demonstrated that PLC had been associated with older age and postmenopausal status. Jung et al. 11 and Jung et al. 12 elucidated that PLC patients tend to be older, to have larger tumor, and to exhibit more axillary LN involvement (higher T and N stages) compared to IDC patients.

Because the incidence of PLC is low, most of the available studies are small retrospective studies or case reports. For this reason, we aim to compare survival outcomes of PLCs with IDCs with large amount of cases and identify prognostic factors that lead to survival differences between the histologic subtypes of breast cancer using the Surveillance, Epidemiology, and End Result (SEER) database. We find 131 available PLC cases, so the statistical result we get will be more accurate.

## Materials and Methods

### Patient selection and data acquisition

The SEER data we use was released in April 2016, which includes data from 18 population‐based registries. The data covers the period from 1973 to 2013. The data of tumor grade, location, and histology are recorded according to the International Classification of Diseases for Oncology Version 3 (ICD‐O‐3). The patient's inclusion criteria were as follows: patients age older than 15, breast cancer (ICD‐O‐3 site code C50), unilateral breast cancer as the first and only cancer diagnosis, diagnosis not obtained from autopsy or a death certificate, there was only one major site, pathologically confirmed invasive ductal carcinoma no other specified (ICD‐O‐3 8500/3) (IDC‐NOS) and pleomorphic lobular carcinoma (ICD‐O‐3 8022/3) with invasion (behavior code ICD‐O‐3 malignant), time of diagnosis from 1990 to 2009.

The items of demographic characteristics included age at diagnosis, the year of diagnosis, marital status, race, laterality, AJCC stage, tumor size, histologic grade, regional LN state, ER status, and PR status. We treated the age of diagnosis as a binary variable that uses the following age group classification: 15–49 years old and 50–85+ years old. We classified year of diagnosis as 1990–1999, 2000–2009.

### Statistical analysis

The clinical and pathological features are compared using Pearson's chi‐square test or Fisher's exact test for classification of nominal data and Cochran–Mantel–Haenszel (CMH) chi‐square test for classification of nominal data. The Kaplan–Meier method is performed to generate 5‐year disease‐specific survival curves, and log‐rank test is performed to compare the difference between curves. In order to eliminate the influencing factors other than the disease type between the two group and get more accurate results, we match every PLCs to IDCs based on the following factors: race, age, year of diagnosis, laterality, PR status, tumor grade, marital status, LN status, tumor stage, ER status, surgery type and radiation. We use propensity score matching method in SPSS and to test match quality to determine the matching balance. All analyzes are performed with the SPSS statistical software, 24.0 version (Armonk, NY, IBM Crop). A two‐sided *P *<* *0.05 is considered to indicate statistical significance.

## Results

### Clinicopathological features of PLC and IDC

According to the criteria we set, we selected 460,240 patients with breast cancer, including 131 PLC patients and 460,109 IDC patients. The tumor demographics and treatment characteristics of histological subtypes are summarized in Table 1. Tumor characteristics with significant statistical differences included histological grade, AJCC stage, and LN status. PLC patients presented with higher grade (grade III: 43.6% vs. 37.3%; *P *<* *0.01) and higher AJCC stage than IDC patients (stage III: 19.1% vs. 11.8% and stage IV: 3.80% vs. 3.40%, respectively; *P *<* *0.01). The PLC patients have more LN‐positive than IDC patients (LN positive: 42.7% vs. 29.9% *P *<* *0.01) and lower median survival months (PLC 84 ± 51.03 vs. IDC 105.2 ± 64.39 *P *<* *0.01) than IDC patients. In the Black race and unmarried population, the proportion of PLC patients was higher than in IDC patients (16.0% vs. 9.1% *P *=* *0.018; 14.5% vs. 12.3% *P *=* *0.023, respectively). The two groups were treated differently. Mastectomy rate was higher in PLCs than in IDCs (54.2% vs. 30.4%; *P *<* *0.01). PLC patients prefer to be less likely to receive radiation therapy than IDC (57.3% vs. 50.9% *P *=* *0.048). We did not find significant difference in laterality, ER, and PR.

**Table 1:** Patient characteristics in PLC compared to IDC.^a^

Variables		IDC, *n* = 460,109 (%)	PLC, *n* = 131 (%)	Total, *n* = 460,240 (%)	*P* b
Median survival months		105.2 ± 64.39	84 ± 51.03	105.01 ± 64.39	**<0.01**
Year of diagnosis	1990–1999	153,948 (33.5)	13 (9.9)	153,961 (33.5)	**<0.01**
	2000–2009	306,161 (66.5)	118 (90.1)	306,279 (66.5)	
Age at diagnosis	15–49	112,714 (24.5)	28 (21.4)	112,742 (24.5)	0.466
	50–85+	347,395 (75.5)	103 (78.6)	347,498 (75.5)	
Race	Black	41,804 (9.1)	21 (16.0)	41,825 (9.1)	**0.018**
	White	379,964 (82.6)	98 (74.8)	380,062 (82.6)	
	Others^c^	38,341 (8.3)	12 (9.2)	38,353 (8.3)	
Marital status	Married	387,230 (84.2)	102 (77.9)	387,332 (84.2)	**0.023**
	Unmarried^d^	56,805 (12.3)	19 (14.5)	56,824 (12.3)	
	Unknown	16,074 (3.5)	10 (7.6)	16,084 (3.5)	
Laterality	Left	233,561 (50.8)	62 (47.3)	233,623 (50.8)	0.485
	Right	226,548 (49.2)	69 (52.7)	226,617 (49.2)	
Grade	I	75,619 (16.4)	1 (0.8)	75,620 (16.4)	**<0.01**
	II	176,421 (38.3)	41 (31.3)	176,462 (38.3)	
	III	166,700 (37.3)	57 (43.6)	166,757 (37.3)	
	Unknown	36,369 (7.9)	32 (24.4)	36,401 (7.9)	
AJCC stage	0	21 (0.0)	0 (0.0)	21 (0.0)	**<0.01**
	I	213,644 (46.4)	34 (26)	213,678 (46.4)	
	II	145,408 (31.6)	59 (45.0)	145,467 (31.6)	
	III	54,398 (11.8)	25 (19.1)	54,423 (11.8)	
	IV	15,827 (3.4)	5 (3.8)	15,832 (3.4)	
	Unknown	30,811 (6.7)	8 (6.1)	30,819 (6.7)	
LN status	Negative	257,886 (56.0)	59 (45)	257,945 (56)	**<0.01**
	Positive	137,444 (29.9)	56 (42.7)	137,500 (29.9)	
	Unknown	64,779 (14.1)	16 (12.2)	64,795 (14.1)	
ER status	Negative	97,679 (21.2)	34 (26)	97,713 (21.2)	0.351
	Positive	303,405 (65.9)	79 (60.3)	303,484 (65.9)	
	Unknown	59,025 (12.8)	18 (13.7)	59,043 (12.8)	
PR status	Negative	137,281 (29.8)	43 (32.8)	137,324 (29.8)	0.748
	Positive	255,245 (55.5)	69 (52.7)	255,314 (55.5)	
	Unknown	67,583 (14.7)	19 (14.5)	67,602 (14.7)	
Surgery type	Mastectomy	139,769 (30.4)	71 (54.2)	139,840 (30.4)	**<0.01**
	Lumpectomy	185,118 (40.2)	46 (35.1)	185,164 (40.2)	
	No surgery	16,730 (3.6)	5 (3.8)	16,735 (3.6)	
	Unknown	118,492 (25.8)	9 (6.9)	118,510 (25.8)	
Radiation	Yes	212,702 (46.2)	49 (37.4)	212,751 (46.2)	**0.048**
	No	234,239 (50.9)	75 (57.3)	234,314 (50.9)	
	Unknown	13,168 (2.9)	7 (5.3)	13,175 (2.9)	

AJCC, American Joint Committee on Cancer; ER, estrogen receptor; PLC, Pleomorphic lobular breast carcinoma; IDC, infiltrating ductal carcinoma; LN, lymph node; PR, progesterone receptor.

a The data are presented as the No. (percentage) of patients unless otherwise indicated.

b *P*‐value of the Chi‐square test to compare the PLC and IDC groups. The value of bold is statistically significant.

c Including American Indian/Alaskan native, Asian/Pacific Islander, and others‐unspecified.

d Including divorced, separated, single (never married), and widowed.

### Comparison of survival outcome between PLCs and IDCs

**Figure 1:**
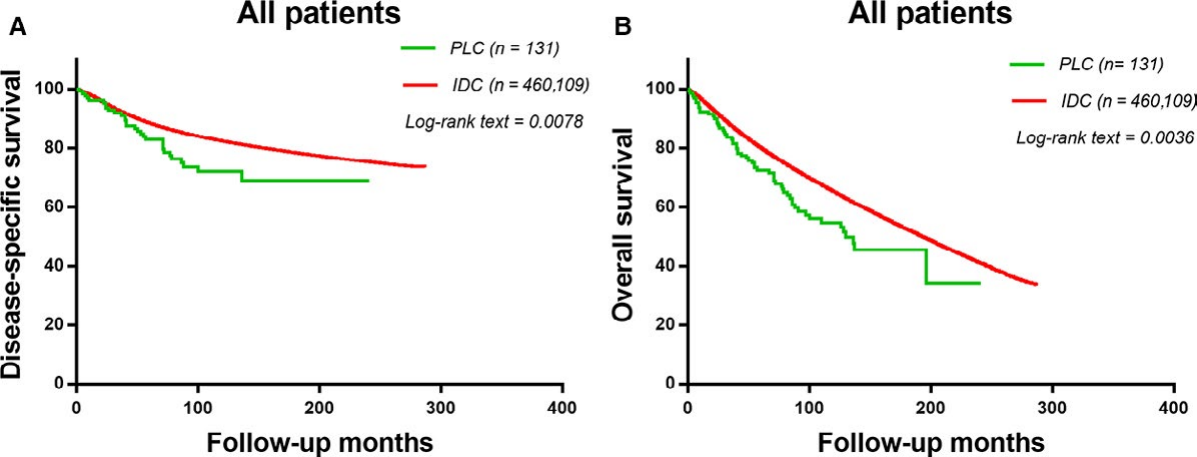
The disease‐specific survival and disease‐overall survival of the two groups. Kaplan–Meier test for disease‐specific survival (*χ* ^2^ = 7.937, *P *=* *0.0078, Fig. 1A) and disease‐overall survival (*χ* ^2^ = 6.619, *P *=* *0.0036, Fig. 1B) to compare PLC patients and IDC patients.

We use Kaplan–Meier plots to evaluate DSS and OS of these two histologic subtypes (Fig. 1A and B). IDCs have better DSS and OS than the overall PLC population (*χ* ^2^ = 7.937, *P *=* *0.0078; *χ* ^2^ = 6.619, *P *=* *0.0036). Five‐year DSS rate of IDC and PLC were 89.0% and 84.7%, respectively. Five‐year OS rate of IDC and PLC were 80.3% and 73.5%, respectively. We used a Cox proportional hazards model to study the effects of baseline characteristics on DSS with univariate and multivariate analysis (Table 2). In the univariate analysis, the prognostic indicators were significantly associated with DSS including patients diagnosed after 2000, patients diagnosed after the age of 50, Black race, higher grade, higher AJCC stage, LN positive, ER/PR negative, no surgery, and radiation. These variables were included in multivariate analysis. The results of multivariate analysis confirmed the prognostic factors of univariate analysis. However, after adjusting for other prognostic factors, the histological type was no longer an independent prognostic factor in multivariate analysis (*P *=* *0.120).

**Table 2:** Univariate and multivariate analysis of disease‐specific survival (DSS)

Variables		Univariate analysis		Multivariate analysis	
		HR (95% CI)	*P*	HR (95% CI)	*P*
Year of diagnosis	1990–1999	Reference	–	Reference	–
	2000–2009	0.840 (0.831–0.848)	**<0.01**	0.923 (0.907–0.938)	**<0.01**
Age at diagnosis	15–49	Reference	**–**	Reference	–
	50–85+	2.120 (2.092–2.147)	**<0.01**	2.330 (2.299–2.361)	**<0.01**
Race	White	Reference	**–**	Reference	**–**
	Black	1.398 (1.377**–**1.419)	**<0.01**	1.244 (1.225**–**1.263)	**<0.01**
	Other^a^	0.694 (0.680**–**0.708)	**<0.01**	0.729 (0.715**–**0.744)	**<0.01**
Marital status	Married	Reference	**–**	Reference	**–**
	Unmarried^b^	1.010 (0.996**–**1.025)	0.159	1.014 (0.999**–**1.029)	0.066
Laterality	Left	Reference	**–**	Reference	**–**
	Right	0.991 (0.982**–**1.001)	0.068	1.000 (0.991**–**1.009)	0.965
Histologic type	PLC	Reference	**–**	Reference	**–**
	IDC	0.691 (0.534**–**0.893)	**<0.01**	0.815 (0.630**–**1.055)	0.120
Grade	I	Reference	**–**	Reference	**–**
	II	1.2947 (1.274**–**1.315)	**<0.01**	1.154 (1.136**–**1.172)	**<0.01**
	III	1.667 (1.642**–**1.693)	**<0.01**	1.365 (1.342**–**1.387)	**<0.01**
AJCC stage	I	Reference	**–**	Reference	**–**
	II	1.350 (1.334**–**1.366)	**<0.01**	1.604 (1.553**–**1.656)	**<0.01**
	III	2.699 (2.662**–**2.736)	**<0.01**	2.423 (2.327**–**2.523)	**<0.01**
	IV	10.760 (10.561**–**10.963)	**<0.01**	4.469 (4.331**–**4.613)	**<0.01**
LN status	Negative	Reference	**–**	Reference	**–**
	Positive	1.833 (1.813**–**1.853)	**<0.01**	1.188 (1.170**–**1.206)	**<0.01**
ER status	Negative	Reference	**–**	Reference	**–**
	Positive	0.804 (0.795**–**0.813)	**<0.01**	0.944 (0.930**–**0.959)	**<0.01**
PR status	Negative	Reference	**–**	Reference	**–**
	Positive	0.758 (0.750**–**0.767)	**<0.01**	0.868 (0.855**–**0.880)	**<0.01**
Surgery type	No surgery	Reference	**–**	Reference	**–**
	Lumpectomy	0.145 (0.142**–**0.148)	**<0.01**	0.604 (0.590**–**0.619)	**<0.01**
	Mastectomy	0.226 (0.221**–**0.230)	**<0.01**	0.690 (0.674**–**0.706)	**<0.01**
Radiation	No	Reference	**–**	Reference	**–**
	Yes	0.590 (0.584**–**0.595)	**<0.01**	0.688 (0.681**–**0.695)	**<0.01**

Multivariate analysis included year of diagnosis, age at diagnosis, race, marital status, laterality, grade, histology, LN status, ER/PR status, surgery type and radiation. HR, hazard ratio; CI, confidence interval; ER, estrogen receptor; PLC, Pleomorphic lobular breast carcinoma; IDC, infiltrating ductal carcinoma; LN, lymph node; PR, progesterone receptor. The bold number of p‐value is statistically significant

a Including American Indian, Alaska Native, Asian, Pacific Islander and others**–**unspecified.

b Including divorced, separated, single (never married), and widowed.

### The survival analysis of the matched group

**Figure 2:**
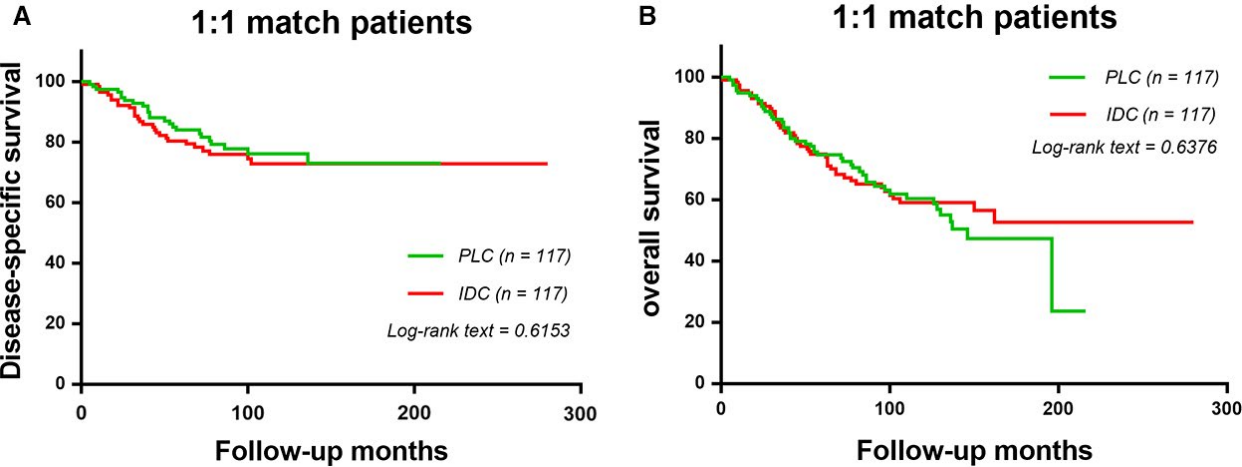
The disease‐specific survival and disease‐overall survival of 1:1 matched groups of PLC patients and IDC patients. Kaplan–Meier test for disease‐specific survival (*χ* ^2^ = 0.2525, *P *=* *0.06153, Fig. 2A) and disease‐overall survival. (*χ* ^2^ = 0.2219, *P *=* *0.6376, Fig. 2B) to compare 1:1 matched groups of PLC patients and IDC patients.

In order to ensure that the difference in survival results is not based on the histological subgroup of demographic and clinical characteristics of the baseline differences, we use the propensity score matching method to perform a 1:1 (IDC: PLC) matched case–control analysis. We obtained 234 patients, of which 117 cases were of PLC, and the remaining117 cases of IDC (Table 3). We used the Cox proportional hazards model for univariate and multivariate analysis to study the effect of baseline characteristics on DSS (Table 4). For matched groups, we find that there is no statistically significant difference in DSS and OS between PLCs and IDCs (Fig. 2A *χ* ^2^ = 0.2525, *P *=* *0.6153, Fig. 2B *χ* ^2^ = 0.2219, *P *=* *0.6376).

**Table 3:** Patient characteristics in matched groups

Variables		IDC	PLC	Total	*P* **–**value^b^
		*N* a = 117(%)	*N* a = 117(%)	*N* a = 234(%)	
Year of diagnosis	1990–1999	9 (7.7)	9 (7.7)	18 (7.7)	1.000
	2000–2009	108 (92.3)	108 (92.3)	216 (92.3)	
Age at diagnosis	15–49	21 (17.9)	21 (17.9)	42 (17.9)	1.000
	50–86+	96 (82.1)	96 (82.1)	192 (82.1)	
Race	White	91 (77.8)	91 (77.8)	182 (77.8)	1.000
	Black	15 (12.8)	15 (12.8)	30 (12.8)	
	Other^c^	11 (9.4)	11 (9.4)	22 (9.4)	
Marital status	Married	100 (85.5)	100 (85.5)	200 (85.5)	1.000
	Unmarried^d^	10 (8.5)	10 (8.5)	10 (8.5)	
	Unknown	7 (6)	7 (6)	14 (6)	
Laterality	Right	61 (52.1)	61 (52.1)	122 (52.1)	1.000
	Left	56 (47.9)	56 (47.9)	112 (47.9)	
Grade	I	1 (0.9)	1 (0.9)	2 (0.9)	1.000
	II	37 (31.6)	37 (31.6)	74 (31.6)	
	III	53 (45.3)	53 (45.3)	106 (45.3)	
	Unknown	26 (22.2)	22 (22.2)	44 (22.2)	
AJCC stage	I	34 (29.1)	34 (29.1)	68 (29.1)	1.000
	II	53 (45.3)	53 (45.3)	106 (45.3)	
	III	21 (17.9)	21 (17.9)	42 (17.9)	
	IV	3 (2.6)	3 (2.6)	6 (2.6)	
	II	6 (5.1)	6 (5.1)	12 (45.1)	
LN status	III	49 (41.9)	49 (41.9)	98 (41.9)	1.000
	Negative	57 (48.7)	57 (48.7)	114 (48.7)	
	Unknown	11 (9.4)	11 (9.4)	22 (9.4)	
ER status	Positive	74 (63.2)	74 (63.2)	148 (63.2)	1.000
	Negative	30 (25.6)	30 (25.6)	60 (25.6)	
	Unknown	13 (11.1)	13 (11.1)	26 (11.1)	
PR status	Positive	63 (53.8)	63 (53.8)	126 (53.8)	1.000
	Negative	40 (34.2)	40 (4.2)	80 (34.2)	
	Unknown	14 (12)	14 (12)	28 (12)	
Surgery type	Mastectomy	67 (57.3)	67 (57.3)	134 (57.3)	1.000
	Lumpectomy	40 (34.2)	40 (34.2)	80 (34.2)	
	No surgery	4 (3.4)	4 (3.4)	8 (3.4)	
	Unknown	6 (5.1)	6 (5.1)	12 (5.1)	
Radiation	Yes	111 (94.9)	111 (94.9)	122 (94.9)	1.000
	No	6 (5.1)	6 (5.1)	12 (5.1)	

AJCC, American Joint Committee on Cancer; ER, estrogen receptor; PLC, Pleomorphic lobular breast carcinoma; IDC, infiltrating ductal carcinoma; LN, lymph node, PR, progesterone receptor.

a The data are presented as the No. (percentage) of patients unless otherwise indicated.

b *P*‐value of the Chi‐square test to compare the PLC and IDC groups.

c Including American Indian/Alaskan native, Asian/Pacific Islander and others‐unspecified.

d Including divorced, separated, single (never married) and widowed.

**Table 4:** Univariate and multivariate analysis of disease‐specific survival (DSS) in matched groups

**Variables**		Univariate analysis		Multivariate analysis	
		HR (95% CI)	*P*	HR (95% CI)	*P*
Year of diagnosis	1990–1999	Reference	–	Reference	–
	2000–2009	0.796 (0.419–1.512)	0.485	0.963 (0.205–4.516)	0.962
Age at diagnosis	15–49	Reference	–	Reference	–
	50–85+	3.756 (1.641–8.596)	**<0.01**	4.455 (1.751–11.338)	**<0.01**
Race	White	Reference	–	Reference	–
	Black	1.738 (0.994–3.039)	0.053	1.723 (0.832–3.571)	0.143
	Other^a^	1.082 (0.540–2.168)	0.823	1.830 (0.843–3.973)	0.127
Marital status	Married	Reference	–	Reference	–
	Unmarried^b^	2.167 (1.176–3.994)	0.013	1.011 (0.424–2.411)	0.980
Laterality	Left	Reference	–	Reference	–
	Right	1.056 (0.705–1.583)	0.790	0.685 (0.404–1.161)	0.160
Group	PLC	Reference	–	Reference	–
	IDC	1.078 (0.720–1.614)	0.715	0.946 (0.617–1.450)	0.798
Grade	I	Reference	–	Reference	–
	II	2.695 (2.602–2.791)	**<0.01**	1.839 (1.775–1.905)	**<0.01**
	III	5.641 (5.453–5.835)	**<0.01**	2.722 (2.628–2.820)	**<0.01**
AJCC stage	I	Reference	–	Reference	–
	II	1.361 (0.792–2.340)	0.265	2.0701.004–4.268)	**0.049**
	III	2.415 (1.325–4.402)	**<0.01**	4.519 (2.001–10.207)	**<0.01**
	IV	11.49 (4.531–29.149)	**<0.01**	9.840 (2.699–35.868)	**<0.01**
LN status	Negative	Reference	–	Reference	–
	Positive	1.287 (0.828–2.001)	0.263	3.512 (1.443–8.549)	**<0.01**
ER status	Positive	Reference	–	Reference	–
	Negative	2.127 (1.350–3.351)	**<0.01**	1.665 (0.466–5.952)	0.433
PR status	Positive	Reference	–	Reference	–
	Negative	2.276 (1.457–3.556)	**<0.01**	0.903 (0.436–1.867)	0.782
Surgery type	No surgery	Reference	–	Reference	–
	Lumpectomy	0.131 (0.056–031)	**<0.01**	0.468 (0.117–1.883)	0.285
	Mastectomy	0.222 (0.100–0.490)	**<0.01**	0.676 (0.182–2.505)	0.557
Radiation	Yes	Reference	–	Reference	–
	No	1.375 (0.636–2.973)	0.418	2.720 (01.084–6.826)	**0.033**

Multivariate analysis included year of diagnosis, age at diagnosis, race, marital status, laterality, grade, histology, LN status, ER/PR status, surgery type, and radiation. HR, hazard ratio; CI, confidence interval; ER, estrogen receptor; PLC, Pleomorphic lobular breast carcinoma; IDC, infiltrating ductal carcinoma; LN, lymph node; PR, progesterone receptor. The value of bold is statistically significant.

a Including American Indian, Alaska Native, Asian, Pacific Islander and others‐unspecified.

b Including divorced, separated, single (never married) and widowed.

### The baseline and survival outcomes of ER‐positive subgroups

**Figure 3:**
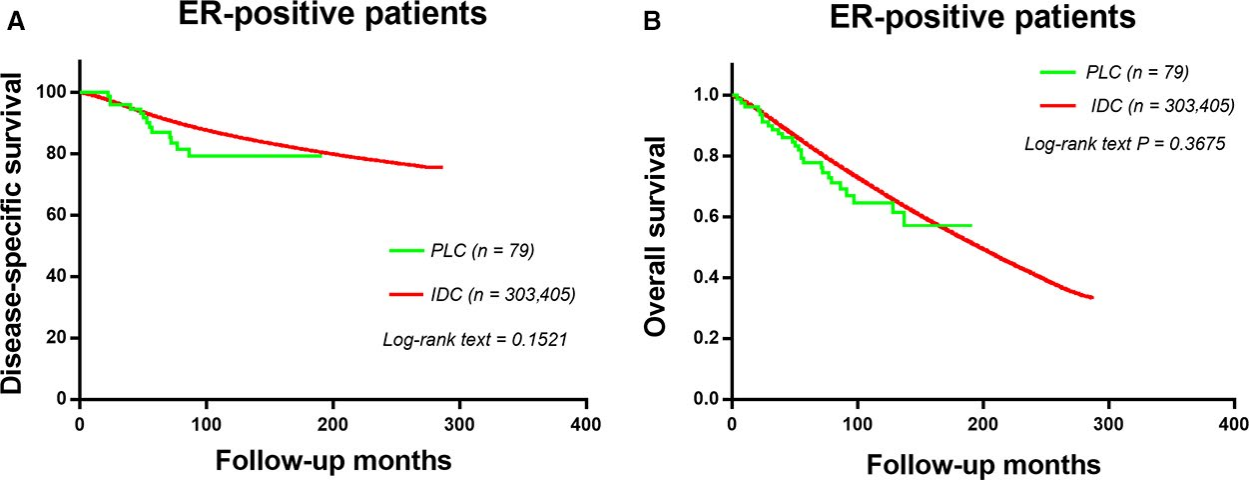
The disease‐specific survival and disease‐overall survival of ER‐positive groups of PLC patients and IDC patients. Kaplan–Meier test for disease‐specific survival (*χ* ^2^ = 1.929, *P *=* *0.1521, Fig. 3A) and disease‐overall survival (*χ* ^2^ = 0.741, *P *=* *0.3674, Fig. 3B) to compare ER‐positive groups of PLC patients and IDC patients.

The proportion of patients with ER positive in PLC and IDC is high and we observed some results in these patients. (Table 5). ER‐positive PLC patients had higher tumor grade, higher AJCC stage, and shorter median survival months than ER‐positive IDC patients. However, there was no statistically significant difference in LN status between ER‐positive PLC patients and ER‐positive IDC patients. Compared with the two groups of ER‐positive patients, there was no statistically significant difference in DSS and OS curves (Fig. 3A *P *=* *0.1521; Fig. 3B, *P *=* *0.3675).

**Table 5:** ER‐positive patient characteristics in PLC compared to IDC^a^

Variables		IDC *n* = 303,405(%)	PLC *n* = 79(%)	Total *n* = 303,484(%)	*P*‐value^b^
Median survival months		105.7 ± 61.154	86.71 ± 44.525	**<0.01**	
Year of diagnosis	1990–1999	93,315 (30.8)	4 (5.1)	93,319 (30.7)	**<0.01**
	2000–2009	210,090 (69.2)	75 (94.9)	210,165 (69.3)	
Age at diagnosis	15–49	67,108 (22.1)	14 (17.7)	67,122 (22.1)	0.420
	50–85+	236,297 (77.9)	65 (82.3)	236,362 (77.9)	
Race	Black	21,190 (7.0)	9 (11.4)	21,199 (7.0)	0.254
	White	256,106 (84.4)	62 (78.5)	256,188 (84.4)	
	Other^c^	26,109 (8.6)	8 (10.1)	26,117 (8.6)	
Marital status	Married	257,816 (85.0)	66 (83.5)	257,882 (85.0)	0.055
	Unmarried^d^	36,195 (11.9)	7 (8.9)	36,202 (11.9)	
Laterality	Left	153,103 (50.5)	38 (48.1)	153,141 (50.5)	0.759
	Right	150,302 (49.5)	41 (51.9)	150,343 (49.5)	
Grade	I	64,652 (21.3)	1 (1.3)	64,653 (21.3)	**<0.01**
	II	138,298 (45.6)	35 (44.3)	138,333 (45.6)	
	III	82,044 (27)	24 (30.3)	78,280 (27)	
	Unknown	18,411 (6.1)	19 (24.1)	18,430 (6.1)	
AJCC stage	I	153,897 (50.7)	26 (32.9)	153,923 (50.7)	**<0.01**
	II	93,087 (30.7)	28 (35.4)	93,115 (30.7)	
	III	31,848 (10.5)	18 (22.8)	31,866 (10.5)	
	IV	9028 (3.0)	3 (3.8)	9031 (3.0)	
	Unknown	15,531 (5.1)	4 (5.1)	15,535 (5.1)	
LN status	Negative	175,444 (57.8)	37 (46.8)	175,481 (57.8)	0.059
	Positive	89,877 (29.6)	33 (41.8)	89,910 (29.6)	
	Unknown	38,084 (12.6)	9 (11.4)	38,093 (12.6)	
PR status	Negative	48,553 (16.0)	12 (15.2)	48,565 (16.0)	0.996
	Positive	246,280 (81.2)	65 (82.3)	246,345 (81.2)	
	Unknown	8572 (2.8)	2 (2.5)	8574 (2.8)	
Surgery type	Mastectomy	89,954 (29.6)	44 (55.7)	89,998 (29.7)	**<0.01**
	Lumpectomy	134,125 (44.2)	29 (36.7)	134,154 (44.2)	
	No surgery	8916 (2.9)	4 (5.1)	8920 (2.9)	
	Unknown	278 (0.1)	0 (0)	278 (0.1)	
Radiation	No	145,644 (48.0)	40 (50.6)	145,684 (48.0)	**<0.01**
	Yes	149,760 (49.4)	32 (40.5)	149,778 (49.4)	
	Unknown	8015 (2.6)	7 (8.9)	8022 (2.6)	

AJCC, American Joint Committee on Cancer; ER, estrogen receptor; PLC, Pleomorphic lobular breast carcinoma; IDC, infiltrating ductal carcinoma; LN, lymph node; PR, progesterone receptor.

a The data are presented as the No. (percentage) of patients unless otherwise indicated.

b *P*‐value of the Chi‐square test to compare the PLC and IDC groups. The value of bold is statistically significant.

c Including American Indian/Alaskan native, Asian/Pacific Islander and others‐unspecified.

d Including divorced, separated, single (never married), and widowed.

## Discussion

With the increasing incidence of breast cancer, the incidence of PLC may also increase. Therefore, it is necessary to obtain more knowledge about the clinical and biological characteristics of the PLC. The factors that limit the current research on PLC are small sample size and short follow‐up time. Therefore, previous studies lack accurate research conclusions about the clinical behavior, prognosis, and treatment strategy of PLC.

This study is the largest analysis of the sample size of PLC. In this study, we retrospectively observed the clinical and pathological characteristics of PLC based on a large number of people. What we found indicated that PLCs were associated with higher histologic grade, higher AJCC stage than IDCs.

Many studies have concluded that the prognosis of PLCs is worse than that of IDCs 2, 13. And our Kaplan–Meier analysis result in DSS and OS show a similar result. However, these findings do not indicate that the PLC itself is an aggressive biological phenotype. Therefore, we adjusted the clinicopathological features and compared DSS and OS with multivariate analysis. The results do not prove that the PLC itself affects the prognosis. Furthermore, after 1:1 matching of PLC with IDC by year of diagnosis, age, race, marital status, tumor grade, laterality, AJCC stage, ER status, surgery type, PR status, LN status and radiation, the PLC displayed almost the same result as IDC in DSS and OS.

Limited information about PLC has been reported in previous studies. Jung and Jung 11, 12 observed that PLCs tend to be older, have larger tumors, and to exhibit more axillary LN involvement (higher T and N stages) than IDCs. In addition, PLCs often display evidence of lymph vascular invasion and a higher proliferative index 13. Most evidence point to PLCs having a lobular origin that develops into a more aggressive phenotype 14, 15. Studies performed earlier showed ER/PR positivity of 9% to 20% in PLC 13, 16. Later publications, however, demonstrate ER/PR positivity of 57% to 96% in PLC 17, 18. Monhollen et al. elucidated that PLC carry with it a higher risk of metastasis and recurrence then IDC.

However, our research has several shortcomings inevitably. First, the current SEER database does not contain the records of adjuvant chemotherapy Ki‐67 expression and endocrine therapy, so we cannot get some important prognostic factors. Second, because Her‐2 was documented in the seer database only after 2010, our data which were collected from 1990 to 2009, did not include that factor. Third, we used propensity score matching method to accomplish our match. In the procedure, 117 IDCs matched with random selection of 117 patients from the patient population may be the reason for the bias sampling, reducing the actual effect of this study.

Compared with IDC, we found that PLC has unique clinicopathological characteristics associated with poor prognosis. However, after we adjust the demographic and clinical pathology factors, this disadvantage is weakened. Improving the clinical and biological understanding of PLC may lead to more personalized and customized treatment for breast cancer patients.

## Conflicts of Interest

The authors declare no conflicts of interest.
